# Rapid and Non-Destructive Detection of Compression Damage of Yellow Peach Using an Electronic Nose and Chemometrics

**DOI:** 10.3390/s20071866

**Published:** 2020-03-27

**Authors:** Xiangzheng Yang, Jiahui Chen, Lianwen Jia, Wangqing Yu, Da Wang, Wenwen Wei, Shaojia Li, Shiyi Tian, Di Wu

**Affiliations:** 1 Jinan Fruit Research Institute, All China Federation of Supply and Marketing Cooperatives, Jinan 250014, China; yangxiangzheng318@163.com (X.Y.); lianwejia@163.com (L.J.); jnbxzx@163.com (W.Y.); wangda19910@163.com (D.W.); flying200807@163.com (W.W.); 2 College of Agriculture & Biotechnology, Zhejiang University, Zijingang Campus, Hangzhou 310058, China; 21816131@zju.edu.cn (J.C.); shaojiali@zju.edu.cn (S.L.); 3 School of Food Science and Biotechnology, Zhejiang GongShang University, Hangzhou 310018, China; tianshiyi@zjgsu.edu.cn

**Keywords:** yellow peach, electronic nose, compression damage, non-destructive, GC–MS

## Abstract

The rapid and non-destructive detection of mechanical damage to fruit during postharvest supply chains is important for monitoring fruit deterioration in time and optimizing freshness preservation and packaging strategies. As fruit is usually packed during supply chain operations, it is difficult to detect whether it has suffered mechanical damage by visual observation and spectral imaging technologies. In this study, based on the volatile substances (VOCs) in yellow peaches, the electronic nose (e-nose) technology was applied to non-destructively predict the levels of compression damage in yellow peaches, discriminate the damaged fruit and predict the time after the damage. A comparison of the models, established based on the samples at different times after damage, was also carried out. The results show that, at 24 h after damage, the correct answer rate for identifying the damaged fruit was 93.33%, and the residual predictive deviation in predicting the levels of compression damage and the time after the damage, was 2.139 and 2.114, respectively. The results of e-nose and gas chromatography-mass spectrophotometry (GC–MS) showed that the VOCs changed after being compressed—this was the basis of the e-nose detection. Therefore, the e-nose is a promising candidate for the detection of compression damage in yellow peach.

## 1. Introduction

Peach (*Prunus persica* L. Batsch) is one of the major fruits worldwide and has an important economic value. Peaches were domesticated in China and are now widely cultivated in the Americas, Europe, and Asia [1]. According to the color of the flesh, peach can be divided into three varieties: white flesh, yellow flesh and red flesh peach. Due to its attractive color, taste, and aroma, yellow peach is very competitive in fruit markets. In comparing the total carotenoid content of yellow flesh and white flesh peach, the former is much higher than the latter. In particular, yellow peaches at harvest have higher β-cryptoxanthin and β-carotene contents [2,3,4].

Fruit ripens and softens quickly after harvest, resulting in a short shelf life [5]. After harvest, the fruit’s metabolism continues, which can cause quality deterioration. Besides this, fruit is susceptible to mechanical damage during the postharvest supply chain, accelerating the deterioration of fruit quality and affecting the commercial value of the fruit [6]. Mechanical wounding during transport and storage can cause fruits such as apples, bananas, and tomatoes to produce large amounts of C_2_H_4_, which, in turn, accelerates fruit ripening and causes quality deterioration [5]. Polashock, et al. [7] found that tomatoes that were mechanically damaged by compression force were less firm, higher in pectin methylesterase (PME) activity, and had greater juice consistency. Li et al. [6] showed that pears with bruise damage were less firm, had a lower the ratio of sugar to acid, and had a higher water loss and disease incidence rate. Mechanical damage also increased the probability of infection with fungi for blueberries, and changed its volatile compounds, e.g., the damaged fruit had higher contents of aldehydes [7].

A major challenge in the fruit supply chain is the rapid detection of fruit damage or decay that occurs during the supply chain steps, such as in the storage room and on the truck. A lack of data on fruit decay causes delays in making the right decisions in a timely manner along the supply chain, such as changing transportation or sale strategies. Acquiring timely information as to whether the fruit has suffered mechanical damage is the key to enhancing information transparency in the fruit supply chain. 

During the whole supply chain of fruits, growers, distributors, sellers, and consumers usually observe fruit decay directly with the naked eye. However, visual observation has disadvantages, such as being subjective, time-consuming, and tedious. The traditional measurement methods for fruit quality are mainly destructive physical/chemical methods, and the measurement process is time-consuming and labor-intensive. Moreover, these methods are based on sampling measurement, which means that only a few samples can be measured, and they so cannot meet the needs of the fruit supply chain industry. Therefore, it is necessary to develop a detection technology that can quickly detect any quality deterioration in the postharvest fruit supply chain.

Currently, the research and application of non-destructive detection methods of fruit freshness and mechanical damage mainly use computer vision, visible and near-infrared spectroscopy, and hyperspectral imaging techniques [8,9]. However, during the supply chain, fruit is usually packed in corrugated fiberboard boxes or plastic containers, and may be covered by additional inner packaging, making it difficult to detect whether the fruit has suffered quality deterioration during the supply chain by visual observation and spectral imaging technologies. In addition, during the fruit supply chain, the environment is usually dark, such as cold storage rooms, carriages, and packages, which makes it difficult to carry out spectral imaging detection. Therefore, there is a need to develop a technology that can detect deterioration in the quality of packaged fruits in a dark environment during the fruit supply chain.

Volatile substances (VOCs) are important indicators in evaluating fruit quality, especially when consumers buy peach fruits and the aroma of the fruit is an important consideration. VOCs, including aldehydes, esters, lactones, terpenes and other substances are abundant in fruits. Fruits produce VOCs during metabolism [10,11], including the postharvest period [12,13,14]. Studies show that the VOCs of fruit have relationships with the fruit quality [15,16,17]. Migliori, et al. [18] found that the VOCs of healthy and decayed tomato fruits were different. Therefore, VOCs are sensitive to the quality deterioration of fruit, and can be used as a characteristic to detect whether the fruit is mechanically damaged or not. 

Electronic nose (e-nose) is a bionic olfactory system that can mimic a human olfactory sensor for VOC recognition [19]. An e-nose system is mainly composed of a sampling system, a gas sensor array and a signal processing system. The sensor array is the core component of the e-nose system that directly affects its sensitivity and accuracy. Metal oxide sensors are widely used in e-nose systems due to their low cost and high sensitivity [20,21,22]. E-nose has been used to detect the early spoilage and deterioration of blueberries [23], citrus [24], and apple juice [25] as well as the maturity of persimmon [26] and citrus [27]. Sanaeifar at al. [11] used an e-nose to predict the total soluble solids, titratable acidity, pH, and the firmness of banana at different shelf-life stages. Hui, et al. [28] successfully used an e-nose system with eight metal oxide semiconductors to distinguish fresh, medium, and aged apples. Di Natale et al. [29] predicted the presence of mealiness and skin damage of apple and storage days of oranges based on the variations in their aromas using e-nose. E-nose has also been used in practical applications, such as the space shuttle [30,31,32]. Feature extraction, measurement denoising, and pattern classification are important for e-nose analysis [33,34,35]. The use of both the steady-state output and temporal or transient information from tin-oxide sensors operating at different temperatures showed its capability of distinguishing various reducing chemicals [36]. In recent years, fluctuation enhanced sensing (FES) was developed to detect gas-phase chemicals [37,38]. Different from conventional approaches that mainly focus on steady-state values of the data that result from the interaction between a chemical sensor and the molecules it detects, FES exploits microfluctuations, which also contain characteristics of different agents, to increase the probability of detection and improve sensitivity and selectivity [39]. FES with a single chemiresistive microsensor has been used for the detection and classification of different gases [40]. In another study, a high-performance chemical and biological agent classification system was proposed to improve the commercial-off-the-shelf (COTS) sensors and other future nanosensors [41]. Currently, the e-nose technology has not been applied to detect compression damage in yellow peach.

For this reason, the aim of this study is to demonstrate the feasibility of applying the e-nose technology to the VOC analysis to predict the levels of compression damage of yellow peach, to discriminate the damaged fruit, and to predict the time after the damage. Multivariate statistical analysis was carried out for data calculation. Moreover, a gas chromatography-mass spectrophotometry (GC–MS) method was used to characterize the possible pattern of VOCs related to compression damage. 

## 2. Materials and Methods

### 2.1. Sample Preparation

Yellow-fleshed peaches (*Prunus persica* L. Batsch cv. Jinxiu) at commercial maturity were harvested from an orchard in Jiaxing (Zhejiang Province, China) on August 10, 2019, and transported to the laboratory in Hangzhou (Zhejiang Province, China) on the same day of harvest. Yellow peach fruit, uniform in size, color, and maturity without mechanical injuries and pests, was selected for the experiments. The selected fruit was divided into three groups, namely, the group of fruit without compression damage (Group 0), the group of fruit compressed by 5 mm (Group I), and the group of fruit compressed by 15 mm (Group II). The compression test to obtain Group I and Group II was performed by the TA.XT Plus Texture Analyzer (Stable Micro Systems, Godalming, UK). A flat-end aluminum round disk probe, 10 mm in diameter, was used, and the compression depths were held constant at 5 mm and 15 mm for Group I and Group II, respectively. The compression speed was 2 mm/s. For Group I and Group II, the e-nose signals of the fruits were collected at 4, 8, and 24 h after the fruit was compressed. The e-nose measurement for Group 0 was at the same time as Group I and Group II. There were 30 fruits for each sampling time, resulting in 90 samples in each group. Before the acquisition of e-nose signal of the fruit, the fruit was sealed in a plastic box for 0.5 h to generate VOCs, and then 10 mL of gas was extracted with a syringe for the e-nose measurement. The used syringe was valve type airtight glass syringe with polytetrafluoroethylene (PTFE) switch (10 mL capacity).

### 2.2. E-Nose Instrument and Data Acquisition

**Figure 1:**
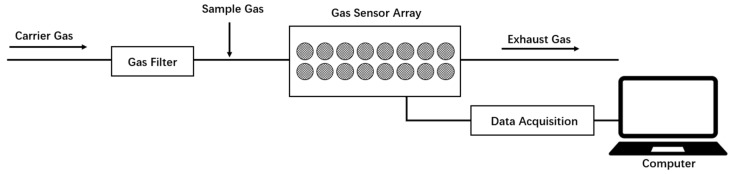
Schematic of the e-nose system.

The e-nose instrument used was developed in-house, and is an updated version of the instrument mentioned in our previous work [42]. Similar to that work [42], this instrument had a gas sensor array, a gas filter and a data acquisition system (Figure 1). In contrast to that work, where the e-nose instrument had 10 sensors in the gas sensor array, this instrument had 14 Figaro TGS series sensors. Their properties are shown in Table 1. Some sensors were selected as their sensitive substances are related to fruit, such as VOC, organic vapors, and organic solvents. These sensors include TGS 2602 (S8), TGS 2620 (S12), TGS 822 (S4) and MQ-138 (S5). Other sensors were selected as they can provide stable signals and are available in the market. The response area of each sensor was used for further data analysis, resulting in 14 variables for each sample. For the parameters of e-nose acquisition, the detection temperature was 40 °C, the acquisition time was 200 s, and the detection and washing flow rates were 0.6 L/min and 2 L/min, respectively. 

### 2.3. Multivariate Data Analysis

The acquisition process of e-nose data did not need to separate the VOCs into individual chemicals, but collected rather the VOCs as a whole. Therefore, the acquired e-nose signals represented the overall situation of the VOCs of the fruit. However, this also made it impossible to determine which e-nose sensors were functioning for the data analysis. Therefore, the e-nose signal needed to be processed by multivariate analysis algorithms to extract key information in the signal for modeling. Partial least squares regression (PLSR) is a widely used multivariate analysis method that is mainly used to build qualitative or quantitative models with discriminative or predictive functions [43]. PLSR finds the fundamental relationships between the variable matrix *Y* (the target attribute) and the variable matrix *X* (the e-nose data). During the calculation of PLSR, latent factors are extracted from the *X* matrix, and then the optimal functions are determined in order to reach the error sum squares. The least squares support vector machine (LS-SVM) is another classic multivariate analysis method with the capability of both linear and non-linear multivariate calibrations. The LS-SVM can solve the multivariate calibration problems in a relatively fast way. To obtain the support vectors, a linear set of equations is used in the LS-SVM instead of a quadratic programming problem [44]. The optimal values of the two main parameters in the LS-SVM model, namely the regularization parameter *γ* and the RBF kernel function parameter *σ*^2^, were determined using the grid-search technique with leave-one-out cross-validation. 

Variable selection is an important step in multivariate data analysis. Among the 14 input variables obtained from the 14 sensors of the e-nose system, not all of them were useful in establishing the model. In this study, two classic variable selection algorithms were considered, namely the successive projections algorithm (SPA) and uninformative variable elimination (UVE). SPA selects variables with minimal redundancy through a projection operation in order to select variables with the minimum collinearity [45,46]. UVE eliminates the variables, in the modeling, with no more information other than noise by calculating the reliability of each variable [47]. 

The established model needed to be evaluated for accuracy and robustness. This process is usually performed using an independent sample set that distinguishes itself from the calibration sample set. When establishing each model in this study, two-thirds of the samples were selected for modeling, and the remaining one-third of the samples were used for verification of the model. The performances of the models, in predicting the levels of compression damage and the time after the damage, were based on the correlation coefficient of calibration (Rc), correlation coefficient of prediction (Rp), root-mean-square error of calibration (RMSEC), root-mean-square error of prediction (RMSEP), residual predictive deviation (RPD), and the absolute difference between the RMSEC and RMSEP (AB_RMSE). The performances of the models in distinguishing the damaged fruit were based on the correct answer rate (CAR), which is the ratio of the number of correctly identified samples to the total number of samples in the model. All calculation for the multivariate data analysis were carried out on MATLAB 2017b software (The MathWorks Inc., Natick, MA, USA).

### 2.4. GC–MS Non-Destructive Measurement

The VOCs from yellow peaches, as a whole, in all three groups at 4, 8, and 24 h after the fruit was compressed were non-destructively acquired and a GC–MS measurement was carried out using a 7860N-5973-C system (Agilent, Wilmington, DE, USA). Tests of each group at each timepoint were repeated four times. The detection procedure was as follows: a single whole fruit was placed in a beaker and sealed with Parafilm for 30 min; then, a SPME fiber (PDMS/DVB, 65 μm, capillary column, Supelco Co., Bellefonte, PA, USA) was used to extract the VOCs of the fruit in the beaker; after 30 min extraction, the fiber that adsorbed VOCs was inserted into the inlet and desorbed at 240 °C for 5 min for the GC–MS analysis. The main parameters of GC–MS were as follows: high-purity helium was used as carrier gas at a flow rate of 1.0 mL/min, and the column model was the DB-WAX quartz capillary column (30 m, 0.25 mm, 0.25 µm); the initial column temperature was 40 °C, and rose to 100 °C at a rate of 3 °C/min and 245 °C at a rate of 5 °C/min. The mass spectrometry parameters were: The ion source was an EI source, ionized at 70 eV of electron energy, the transfer temperature was 250 °C, and the ion source temperature was 230 °C. 

## 3. Results

### 3.1. E-Nose Response of Peach Fruit

Table 2 shows the average response of the e-nose signal for samples with different levels of compression damage (0, 5, and 15 mm) and different times after compression (4, 8, and 24 h). It can be seen that different levels of compression damage and different time after compression made the e-nose signals different. When the e-nose signal was acquired at 4 h after the compression, the signal of the samples with different levels of compression damage was similar (Figure S1). When the time was 24 h, there were obvious differences in the e-nose signal between the different levels of compression damage, especially for sensors 1, 2, 5, 6, 8, 9, and 11, whose e-nose signals were positively correlated with the level of damage that the samples suffered (Figure S1). However, it can be seen from Table 2 that it was difficult to clearly distinguish between different levels of compression damage or the time after damage through a particular sensor. In particular, due to the differences among individual peach fruit samples, even if they suffered the same compression damage and were stored for the same time after being compressed, there was a large difference between their e-nose signals, such as the variance of the signal being large, which is very normal. Due to the positioning of the tree that each peach fruit was picked from, the location of the tree, the cultivation method, and the soil and other environmental conditions are different for different fruits, resulting in differences in the VOCs after each fruit suffered compression damage. Therefore, it is necessary to use multivariate data analysis to analyze the signals of all 14 sensors together and to establish chemometric models. 

### 3.2. Detection of Levels of Compression Damage

In order to study whether the e-nose technology can be used to detect the levels of compression damage to peach fruits, the e-nose signals of all 14 sensors were used as independent variables in the model, the level of compression damage was used as a dependent variable, and PLSR and LS-SVM models were established, respectively. There were 30 samples for each compression level and each time after the compression; there were three levels of compression damage (0, 5, and 15 mm) and three kinds of time (4, 8, and 24 h), resulting in a total of 270 samples (30 samples × 3 levels × 3 times). Among them, 180 samples were selected by a sample set partitioning based on the joint X-Y distance (SPXY) algorithm [48] for the model calibration and the remaining 90 samples as the independent samples for prediction. Besides this, only the models for the samples stored for 4, 8, and 24 h after compression were established, respectively. For these models, 90 samples were used to establish the model (30 samples × 3 levels), and among them, 60 samples were selected by SPXY for calibration and the remaining 30 samples for prediction. The results of these models are shown in Table 3. 

When samples from all three kinds of time were used for modeling, neither the PLSR nor the LS-SVM model could predict the level of compression damage suffered by the fruit. Only the fruit at 4 or 8 h after compression damage occurred was used for modeling, and still neither of the two modeling algorithms could obtain effective models. Only when the fruit at 24 h after compression damage occurred was used for modeling did, the LS-SVM algorithm obtain a good model, with an RPD value reaching 1.745 and a Rp value of 0.822. However, the corresponding PLSR model still could not obtain good prediction results. This shows that, after the yellow peach suffered compression damage, in a short period of time, such as 4 or 8 h, the changes in its VOCs were not enough to be reflected by the e-nose signal. At 24 h after damage, the VOCs of the yellow peach fruit could be used to establish an e-nose detection model, but the LS-SVM algorithm, capable of being used for nonlinear modeling, must also be used. Only the PLSR algorithm, which can be used for linear modeling, was still unable to predict the level of compression damage suffered by the peach fruit. 

Two variable selection algorithms were further used to improve the prediction accuracy of the models. The variable selection calculation was performed for 24 h samples first. This is because when full variables were considered for modeling, only the LS-SVM model based on 24 h samples obtained good results. Two algorithms, UVE and SPA, were used for variable selection, and ten variables and two variables were obtained from all 14 variables, respectively. The selected variables were used for modeling, and the results are shown in Table 3. 

Through the calculation of UVE, the established UVE-LS-SVM model has better prediction accuracy than the full-variable LS-SVM model, in which the RPD value increased by 22.58%, while the RMSEP value decreased by 18.41%. However, the PLSR model, based on the variables selected by UVE, could not improve the accuracy of the model. When the variables selected by SPA were used to establish the models, the accuracy of the prediction sample set was similar to that of the full-variable LS-SVM model, but the calibration sample set was significantly worse. It shows that although the calculation of SPA reduced the number of variables from 14 to 2, which is significant, these two variables were not enough to represent the overall VOCs in order to detect the level of compression damage. Besides the 24 h samples, UVE and SPA were also calculated for the 4 and 8 h samples, but no improvement was achieved.

### 3.3. Discrimination of Damaged Fruit

The levels of compression damage detection results show that the best UVE-LS-SVM model had the highest RPD, at 2.139, indicating that the model is usable for screening [49]. Besides the detection of the damage level, the discrimination of the damaged fruit is also important for the industry. Similar to Section 3.2, all 14 sensors were used for modeling, based on four kinds of sample sets, namely, all-time samples, 4 h samples, 8 h samples, and 24 h samples, and the results are shown in Table 4. When PLSR was used for modeling, all four kinds of sample sets failed to discriminate between samples, aalthough, from the perspective of overall accuracy, these models had a certain ability to distinguish the samples from the calibration sample set and the prediction sample set. However, looking specifically at the discrimination results for the healthy fruit and the damaged fruit, most samples from the calibration sample set and all the samples from the prediction sample set for the healthy fruit were judged to be the damaged samples. This shows that, in fact, these PLSR models had no ability to distinguish fruit damage. When the LS-SVM algorithm was used for modeling, the results of the LS-SVM models based on the all-time samples, 4 h samples, and 8 h samples were not good, which was similar to the results of the detection of levels of compression damage. Most healthy samples in the prediction sample set were judged to be damaged samples. When the 24 h samples were used for modeling, good discrimination results were obtained, not only from the overall discrimination results, in which the CARs of the calibration sample set and prediction sample set were 96.67% and 86.67%, respectively, but also from the healthy and damaged fruits, with most samples being correctly distinguished. Although looking at the overall discrimination results, the LS-SVM model established, based on the 24 h samples, had not improved much, as compared to the PLSR and LS-SVM models that were established based on other sample sets (CARS improved from between 60% and 80% to over 80%), but the discrimination results of healthy samples and damaged samples were much better. The feasibility of improving the accuracy of damage discrimination through variable selection was further evaluated. The results are shown in Table 4. Only the UVE-LS-SVM model achieved better discrimination accuracy. Among them, the calibration sample set reached 100% CAR, and the prediction sample set reached more than 90% CAR. Moreover, all damaged fruits were distinguished 100% of the time. The above results show that, when the compression damage occurred for 24 h, the VOCs of the yellow peach fruit could be used to determine whether the fruit had suffered compression damage. 

### 3.4. Prediction of Time after Compression Damage 

Predicting when compression damage occurred is very important in order to discover the cause of the compression damage and to optimize solutions for the fruit supply chain. Based on three samples sets, namely, the two sample sets of fruits compressed by 5 and 15 mm, respectively, and their combined sample set, the prediction models for the occurrence time of compression damage (4, 8, and 24 h) were established, respectively. The former two sample sets had 90 samples (30 samples × 3 times), respectively; 60 of them were selected by SPXY for calibration and the remaining 30 samples for prediction. The combined sample set had 180 samples, in which 120 of them were selected by SPXY for calibration and the remaining 60 samples for prediction. The results are shown in Table 5. When the fruit compressed by 5 mm was used for modeling, neither the PLSR algorithm nor the LS-SVM algorithm could establish a model that could be used to predict the occurrence time of compression damage. On the contrary, when the fruit compressed by 15 mm was used for modeling, both the PLSR and LS-SVM models obtained good prediction with RPD values of 1.959 and 2.114, respectively. Nevertheless, the PLSR model had a poorer calibration result than the LS-SVM model. When the fruits with two levels of compression damage were used for modeling together, both the PLSR and LS-SVM models had poor results. The variable selection of the e-nose data from the fruit compressed by 15 mm was further performed, and the results are shown in Table 5. It can be seen that neither the UVE nor the SPA algorithm could improve the prediction accuracy of the models. The above results show that, when the fruit suffered severe compression damage, such as 15 mm, the differences in the VOCs could be used to predict when the compression damage occurred. On the contrary, if the fruit suffered only light compression damage, such as 5 mm, the change in the VOCs of the fruit might not be enough to be detected by the e-nose system. Moreover, when the fruit suffered light mechanical damage, the fruit had the ability to heal itself [50,51], resulting in less difference of VOCs between the damaged fruit and healthy fruit, making it more difficult to detect whether the fruit had suffered mechanical damage. 

### 3.5. GC–MS Analysis

GC–MS technology was used to analyze the changes of VOCs in the whole yellow peach fruit after compression damage. By analyzing the GC–MS results of yellow peach fruit in nine sample sets with three levels of compression damage and three kinds of time after compression, the VOCs of fruit were mainly esters, alkanes, benzene rings, phenols, and terpenes. Among them, esters were the most dominant, and their relative content was also the highest, accounting for 32.60–81.39%. After 4 h of compression damage, a total of 35 VOCs were detected, including esters (13), alkanes (10), benzene rings (5), terpenes (3), alcohol (1), phenol (1), ether (1), and ketone (1). After 8 h of compression damage, a total of 33 VOCs were detected, including esters (17), alkane (9), benzene rings (3), phenol (1), alcohol (1), thiazole (1), and pyrimidine (1). After 24 h of compression damage, a total of 34 VOCs were detected, including esters (19), alkane (9), terpene (1), benzene ring (1), phenol (1), alcohol (1), ether (1), and ketone (1). 

**Figure 2:**
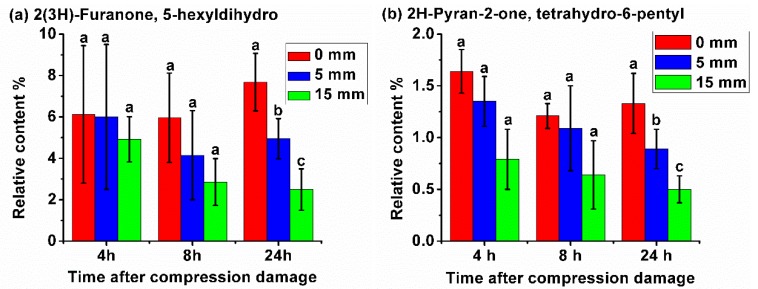

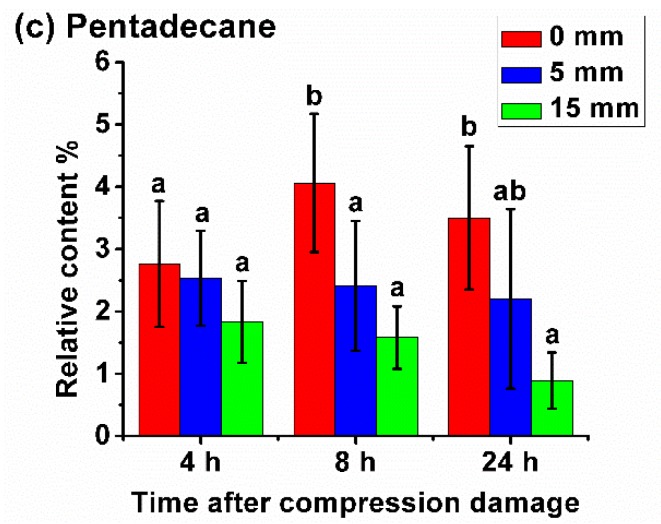
(**a**–**c**) Relative content of three volatile substances (VOCs) decreased with the increase of the level of compression damage.

Further analysis of the change in the content of each VOC was performed. It can be seen that there was a certain change between the content of some VOCs from the samples with different levels of compression damage and different times after damage. Among them, the relative content of three VOCs decreased with the increase in the level of compression damage (Figure 2). As shown in Figure 2a, for 5-hexyldihydro-2(3*H*)-furanone, 4 h after compression damage, its relative content was not significantly different from the samples with different compression damages. At 8 h, its content in the damaged samples was lower than that of the samples without compression damage, and the greater the compression damage, the lower the content; however, there was no significant difference between them. At 24 h, there was significant differences between the three compression treatments. In addition, when the fruit was subjected to 15 mm compression, its content decreased with time. For tetrahydro-6-pentyl-2*H*-pyran-2-one, as shown in Figure 2b, as the level and time of compression damage increased, its relative content decreased. While there was no significant difference between the samples of different compression treatments at 4 and 8 h, there was a significant difference between the samples of three compression treatments at 24 h. For Pentadecane, as shown in Figure 2c, as the level and time of compression damage increased, its relative content also decreased, and at 8 and 24 h, there was a significant difference between the healthy and damaged samples. 

**Figure 3:**
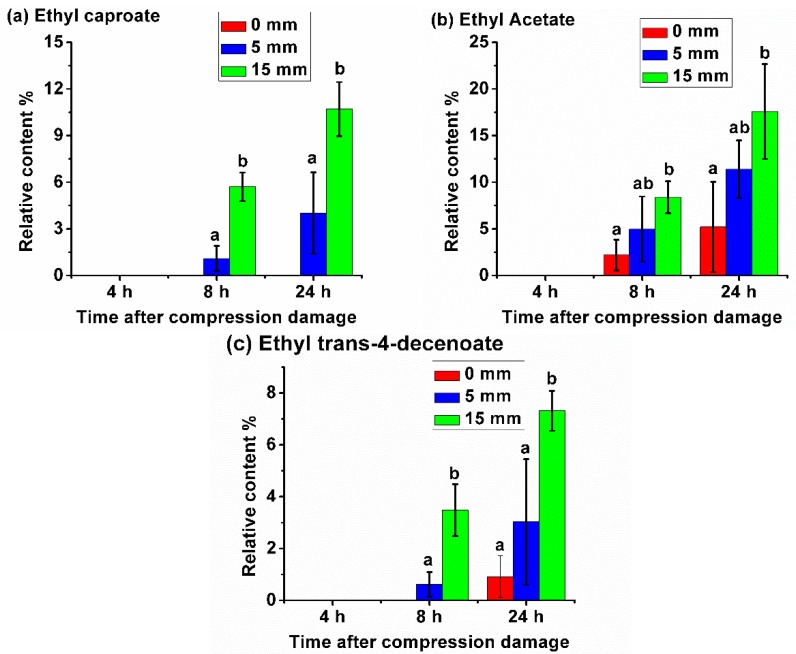
(**a**–**c**) Relative content of three VOCs increased with the increase of the level of compression damage.

There were another three VOCs, whose relative content increased with the increase in the level of compression damage (Figure 3). For ethyl caproate, as shown in Figure 3a, nothing was detected in all three compression treatments at 4 h. Over time, the content of this VOC in the damaged fruit increased continuously, and there was a significant difference between the samples compressed by 5 and 15 mm. This VOC was never detected in the samples without compression damage at all storage times. For ethyl acetate, as shown in Figure 3b, nothing was detected in all three compression treatments at 4 h. As time increased, its content in healthy samples gradually increased, but its content in the samples that suffered compression damage increased more rapidly, and the more severe the compression, the higher the content increased. At 8 and 24 h, there was a significant difference between the healthy samples and the samples compressed by 15 mm. For ethyl *trans*-4-decenoate, as shown in Figure 3c, nothing was detected in all three compression treatments at 4 h. For healthy samples, only a small amount was detected at 24 h. For the samples compressed by 5 mm, only a small amount was detected at 8 h, and the content was more obvious at 24 h. For the samples compressed by 15 mm, the content was already evident at 8 h and significantly increased at 24 h. 

**Figure 4:**
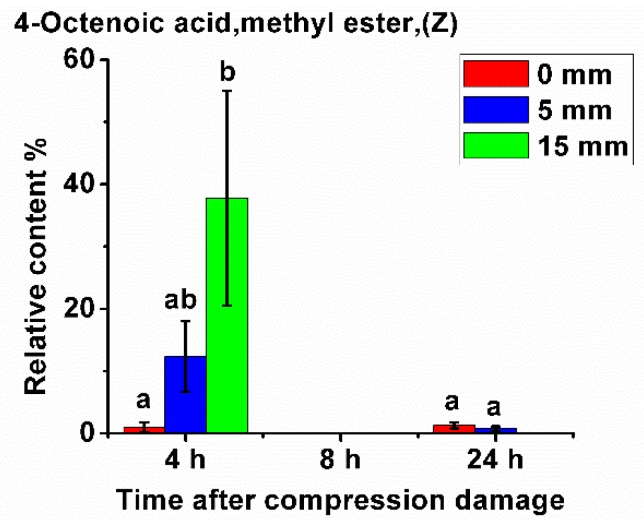
Relative content of 4-(Z)-octenoic acid methyl ester after compression damage.

In addition, as shown in Figure 4, after 4 h of compression damage, 4-(*Z*)-octenoic acid methyl ester was produced in the fruit, and its content was related to the level of compression damage. The more severe the compression, the higher its content. Its content in the fruit without compression damage was very low. 

However, no 4-(*Z*)-octenoic acid methyl ester was detected after 8 h of compression damage, and the content was also very low at 24 h. This shows that a large amount of 4-(*Z*)-octenoic acid methyl ester was produced shortly after compression damage occurred, resulting in a significant increase in its content; however, with the increase of time after damage, its content decreased. This may be due to the metabolic process of degradation in 4-(*Z*)-octenoic acid methyl ester, in the fruit.

To show which of the 14 e-nose sensors responds best to a given volatile compound in the case of yellow peach, the relative contents of seven volatile compounds determined by GC-MS and shown in Figure 2, Figure 3, and Figure 4 were correlated with the responses of the e-nose sensors by PLSR calculation. The contents of these volatile compounds changed after being compressed. Therefore, these compounds were selected for calculation. The regression coefficients of PLSR models show that sensors 10, 8 and 6 were important to predict the content of 5-hexyldihydro-2(3*H*)-furanone, sensors 8, 9 and 13 were important to predict the content of tetrahydro-6-pentyl-2*H*-pyran-2-one, sensors 8, 13 and4 were important to predict the content of pentadecane, sensors 8, 2 and 11 were important to predict the content of ethyl caproate, sensors 8 and 2 were important to predict the content of ethyl acetate, sensors 8 and 2 were also important to predict the content of ethyl *trans*-4-decenoate, and sensors 13, 8 and 4 were important to predict the content of 4-(*Z*)-octenoic acid methyl ester. 

## 4. Discussion

Previous studies found that the VOCs of fruit changed during its development and ripening [15,52]. Besada et al. [15] identified VOCs associated with immature stages, intermediate maturity stages and ripening and correlated VOCs with loquat aroma/flavor. Song et al. [52] characterized VOCs of jujube fruits at different ripening stages. Other studies also found that postharvest treatments can affect VOC production in fruit [53,54]. According to Lu et al. [53], although the aroma production of apple fruit during storage was significantly inhibited by 1-MCP treatment of 1.0 μL L^−1^, (1-MCP 0.6 +Ca) treatment promoted VOC production by improving the enzyme activities of alcohol dehydrogenase (ADH), pyruvate decarboxylase (PDC) and aromatase-related acyltransferase (AAT). The results of Echeverría et al.’s [54] work show that VOCs of Fuji apples kept under different storage conditions were different. In addition, pathogenic fungal diseases and pests were found to affect the VOCs of fruit [55,56,57]. Pan et al. [55] identified several key VOCs for infection treatment of strawberry fruit on day 2. According to Wen et al. [56], the VOCs of citrus fruits infested by *Bactrocera dorsalis* (Hendel) were different. Liu et al. [57] found that changes in VOCs in fungi-inoculated peaches were correlated with total amounts and species of fungi. In our work, the e-nose and GC–MS results showed that the VOCs of yellow peach fruit changed after being compressed. The difference of VOCs and their content in samples with different compression levels and time was the reason that the e-nose technology could be used for the rapid and non-destructive detection of compression damage in yellow peach. Considering that fruit is usually stored in packaging boxes during supply chain, the detection of mechanical damage, based on VOCs, will be more suitable than optical detection technology for application in the fruit supply chain. 

By using an array of sensors to simulate olfactory, e-nose systems have been used to detect fruit quality. Li et al. [58] used e-nose to predict quality of Chinese bayberry, resulting in an accuracy of 95%. Zhang et al. [26] assessed soluble solids content of persimmon fruit picked on different dates using e-nose technique. An average prediction accuracy of 91.36% was obtained by support vector machine models. Our previous study found that the VOCs of peach fruit could be used to rapidly and non-destructively detect fruit decay [59]. The CAR value of the best model to classify decayed fruit was 95.83% (94.64% for healthy samples and 100.00% for decayed samples). The RPD value of the best model to predict the storage days of peach fruit was 9.283. Other studies were also carried out using e-nose to detect plant quality. Rusinek et al. [21] used an e-nose technique to identify the loss in the rapeseed quality during storage. A new three-parameter method based on the impregnation time, cleaning time, and maximum response was proposed to electronic fingerprints, which could distinguish the rapeseed samples infected with microflora. In another study, this three-parameter method was used to assess the quality of cold-pressed rapeseed oil and then to determine the pre-pressing treatment of rapeseed [22]. The above studies show that e-nose was an efficient, rapid and non-destructive method to analyze the VOCs of samples and to determine the quality of samples. Especially for the mechanical damage of fruit, previous works have been carried out to detect impact damage using e-nose technique. Demir et al. [60] classified fresh blueberries with impact damage, based on their VOCs during storage. E-nose data of blueberries were acquired on days 0, 2, 10, 17 and 24 after the impact treatment. Discriminant function analysis was used to classify samples, resulting in correct classification rates of 0, 100, 100, 100 and 100% for days 0, 2, 10, 17 and 24, respectively. Volatiles of fruit on day 0 had no significant difference. In another study, Ren et al. [61] determined the damage degree of apples dropped from different heights using e-nose, coupled with multivariate statistical analyses. Back-propagation neural network model obtained good classification of damaged apples. In our study, based on the variation of VOCs in yellow peach fruits, the non-destructive detection of compression damage was performed using e-nose technology. It should be noted that, among the various mechanical damages that the fruit suffered during the postharvest supply chain, compared to the impact and vibration damages, compression damage usually causes less damage to the fruit, and so it is more difficult to carry out the detection of compression damage through the VOCs in fruit. Moreover, besides the classification of damaged fruits like previous work did, the levels of compression damage were also detected based on the e-nose data of peach fruit in this work. The detection of damage levels can provide more detailed information on the damage, which is important for the fruit industry. 

In addition, previous works on the detection of mechanical damage of fruit mainly focused on the classification of damaged fruit or detection of damage levels [60,61]. As it is impossible to know when the compression damage occurred in the fruit supply chain, and because the fruit continues to undergo metabolism after compression damage, there are continuous changes in the VOCs of fruit. Therefore, this study also carried out a comparative study of models established based on the samples at different times after compression damage, so that the established models are more in line with the actual situation.

To further develop a dedicated e-nose detection instrument, it is necessary to analyze the changing rules and mechanisms of the VOCs in the fruit, as a whole, caused by mechanical damage. This study performed a non-destructive GC–MS analysis of the VOCs of yellow peach, and the results show that the compression damage did cause changes in the VOCs of the fruit. Previous studies also used GC–MS technology to find differences in the VOCs of fresh-cut broccoli with different freshness levels [62], and found that the VOCs of strawberry fruit also changed after being infected with the disease [55]. However, in these studies, the fruit was cut and blended for VOC extraction and GC–MS analysis. Nevertheless, the VOCs released after the fruit is damaged during the supply chain are from the whole fruit, not the blended pulp. Therefore, the acquisition of both the e-nose signal and the GC–MS data in this study was from the whole fruit, not the pulp. 

Chemometric analysis is an important step for e-nose study and application. For the two multivariate analysis algorithms used in this study, the results of the LS-SVM models were better than the PLSR models. This is similar to previous research [59,63], indicating that the nonlinear modeling algorithm is more suitable for the data analysis of the e-nose instruments based on metal oxide sensors. 

Before the practical application, some other issues need to be solved based on this primary study. First, this study only simulated the compression damage of yellow peach fruit. During the supply chain, the fruit suffers from a variety of mechanical damages. These mechanical damages can also cause a combination change in the VOCs of the fruit. Therefore, further research is needed for these mechanical damages. Second, in this study, the ambient temperature for e-nose signal collection was 20 °C. Nevertheless, a cold chain environment was commonly considered during the fruit supply chain, and the released VOCs of fruit at different temperatures were different. Therefore, in further research, the effect of different temperatures on the detection of fruit mechanical damage needs to be considered. Thirdly, more fruit samples from different harvest years, orchards, varieties, planting pattern and climates are required to make the models more practically applicable. In addition, the small size and low cost of e-nose instruments are important. The former will facilitate the installation in transport boxes, cold storage rooms, refrigerators, and carriages, while the latter will facilitate the industrialization of the instruments.

## 5. Conclusions

This study investigated the feasibility of using the e-nose technology for the rapid and non-destructive detection of the compression damage of yellow peach. The results show that, at 24 h after the compression damage, the levels of compression damage could be predicted, and the best UVE-LS-SVM model had the highest RPD of 2.139, whereas the models established based on the e-nose data, collected at 4 and 8 h after the damage were not good. The results of the discrimination of damaged fruit also show that the VOCs of the yellow peach fruit could be used to determine whether the fruit has suffered compression damage when the compression damage occurred for 24 h. The CAR for prediction was 93.33%, and all damaged fruit was correctly identified. The time when the compression damage occurred could be predicted when the fruit suffered severe compression damage, such as 15 mm (which is used in this study). Light compression damage, such as 5 mm (which is used in this study) could not cause a sufficiently large change of VOCs to be used to predict the time after damage using e-nose technology. The results of GC–MS identified that three VOCs decreased, and three other VOCs increased, with the increase of the level of compression damage, showing that the VOCs of yellow peach fruit changed after being compressed, which was the basis for e-nose to detect the compression damage of yellow peach. This study opens up an attractive prospect for using the e-nose technology in the non-destructive detection of fruit damage during the postharvest supply chain. 

## Figures and Tables

**Table 1:** Gas sensor array and its properties.

Sensor Number	Sensor Model	Sensitive Substances
S1	TGS 826	Ammonia, amines
S2	MQ-136	Hydrogen sulfide, sulfide
S3	TGS 821	Hydrogen
S4	TGS 822	Alcohol, organic solvents
S5	MQ-138	Toluene, acetone, ethanol, formaldehyde, hydrogen, and other organic vapors
S6	MQ-4	Methane, biogas, natural gas
S7	TGS 813	Methane, propane, isobutane, natural gas, liquefied gas
S8	TGS 2602	Cigarette smoke, cooking odor, VOC, ammonia, hydrogen sulfide, alcohol
S9	MQ-5	Butane, propane, methane, liquefied gas, natural gas, gas
S10	TGS 2610	Liquefied petroleum gas, combustible gas, propane, butane
S11	MQ-2	Propane, smoke, combustible gas
S12	TGS 2620	Carbon monoxide, ethanol, organic solvents, other volatile gases
S13	TGS 2600	Smoke, cooking odor, hydrogen, carbon monoxide, air pollutants
S14	TGS 2611	Methane, natural gas

**Table 2:** Mean values and standard deviations of electronic nose (e-nose) responses of yellow peach from three groups at 4, 8, and 24 h after the fruit was compressed. Fruits without compression damage were in Group 0. Fruits compressed by 5 mm were in Group I. Fruits compressed by 15 mm were in Group II.

Sensor Number		0 mm		5 mm		15 mm	
		Mean Value	Standard Deviation	Mean Value	Standard Deviation	Mean Value	Standard Deviation
1	4 h	1.389	1.125	1.240	0.697	1.293	0.686
	8 h	2.122	1.308	1.641	0.834	1.293	0.686
	24 h	1.303	0.495	1.544	0.971	2.539	1.083
2	4 h	1.410	1.288	1.462	0.991	1.310	0.949
	8 h	2.040	1.634	1.332	1.004	1.310	0.949
	24 h	1.235	0.603	1.613	1.273	2.299	1.447
3	4 h	0.126	0.078	0.085	0.069	0.101	0.070
	8 h	0.088	0.056	0.108	0.081	0.101	0.070
	24 h	0.106	0.076	0.106	0.091	0.111	0.083
4	4 h	0.868	0.423	0.712	0.294	0.720	0.271
	8 h	0.803	0.277	0.739	0.418	0.720	0.271
	24 h	1.059	0.952	0.892	0.367	1.223	0.504
5	4 h	0.207	0.114	0.257	0.178	0.248	0.151
	8 h	0.290	0.238	0.265	0.188	0.248	0.151
	24 h	0.229	0.127	0.304	0.196	0.462	0.243
6	4 h	1.752	0.536	1.650	0.377	1.628	0.313
	8 h	1.804	0.748	1.641	0.423	1.628	0.313
	24 h	1.388	0.206	1.607	0.359	1.897	0.467
7	4 h	0.576	0.315	0.474	0.082	0.504	0.078
	8 h	0.564	0.394	0.521	0.167	0.504	0.078
	24 h	0.612	0.564	0.599	0.426	0.713	0.247
8	4 h	0.749	0.728	0.570	0.403	0.885	0.659
	8 h	1.635	1.418	1.108	0.540	0.885	0.659
	24 h	1.079	1.002	1.116	0.960	2.366	1.503
9	4 h	1.577	0.977	1.493	0.649	1.362	0.560
	8 h	1.844	1.188	1.390	0.629	1.362	0.560
	24 h	1.199	0.283	1.418	0.614	1.793	0.751
10	4 h	0.939	0.294	0.836	0.190	0.855	0.201
	8 h	0.937	0.325	0.968	0.360	0.855	0.201
	24 h	1.040	0.864	0.937	0.231	1.240	0.331
11	4 h	1.028	0.876	0.949	0.506	0.921	0.509
	8 h	1.299	0.922	0.978	0.544	0.921	0.509
	24 h	0.780	0.288	1.076	0.679	1.706	0.801
12	4 h	1.621	0.840	1.358	0.545	1.431	0.517
	8 h	1.466	0.567	1.549	0.892	1.431	0.517
	24 h	1.795	1.442	1.645	0.617	2.260	0.980
13	4 h	1.866	0.938	1.493	0.554	1.579	0.491
	8 h	1.612	0.586	1.682	0.899	1.579	0.491
	24 h	2.139	1.858	1.821	0.645	2.429	0.993
14	4 h	1.423	0.442	1.242	0.301	1.325	0.300
	8 h	1.428	0.618	1.457	0.564	1.325	0.300
	24 h	1.542	1.377	1.410	0.371	1.884	0.563

**Table 3:** Detection of levels of compression damage.

Time	Variable	Calibration	Calibration			Prediction				AB_RMSE
			Rc	Rc^2^	RMSEC	Rp	Rp^2^	RMSEP	RPD	
all	all	PLSR	0.367	0.135	6.068	0.301	0.078	5.424	1.041	0.644
4 h	all	PLSR	0.205	0.042	6.184	0.023	−0.069	6.255	1.000	0.071
8 h	all	PLSR	0.171	0.029	6.497	−0.139	−0.011	5.690	0.995	0.807
24 h	all	PLSR	0.384	0.147	5.907	0.790	0.162	5.485	1.099	0.422
all	all	LS-SVM	0.611	0.354	5.243	0.430	0.157	5.154	1.096	0.089
4 h	all	LS-SVM	1.000	1.000	0.005	0.565	0.267	5.167	1.210	5.162
8 h	all	LS-SVM	0.915	0.775	3.128	0.373	0.049	5.308	1.067	2.180
24 h	all	LS-SVM	0.853	0.651	3.778	0.822	0.670	3.455	1.745	0.323
24 h	UVE	PLSR	0.394	0.155	5.881	0.820	0.230	5.285	1.141	0.596
24 h	UVE	LS-SVM	0.966	0.922	1.790	0.888	0.768	2.819	2.139	1.029
24 h	SPA	PLSR	0.642	0.412	4.907	0.832	0.637	3.525	1.710	1.382
24 h	SPA	LS-SVM	0.660	0.434	4.813	0.839	0.675	3.404	1.771	1.409

**Table 4:** Discrimination of damaged fruit.

Time	Variable	Calibration	All		Health		Damaged	
			Calibration	Prediction	Calibration	Prediction	Calibration	Prediction
all	all	PLSR	63.89%	74.44%	3.00%	0.00%	100.00%	100.00%
4 h	all	PLSR	63.33%	76.67%	34.78%	0.00%	91.89%	100.00%
8 h	all	PLSR	60.00%	80.00%	37.50%	0.00%	88.89%	79.17%
24 h	all	PLSR	71.67%	63.33%	26.32%	0.00%	97.56%	100.00%
all	all	LS-SVM	87.22%	77.78%	38.81%	84.96%	65.22%	46.27%
4 h	all	LS-SVM	95.00%	66.67%	91.30%	28.57%	97.30%	78.26%
8 h	all	LS-SVM	100.00%	76.67%	100.00%	33.33%	100.00%	87.50%
24 h	all	LS-SVM	96.67%	86.67%	94.74%	100.00%	97.56%	78.95%
24 h	UVE	PLSR	71.67%	63.33%	10.53%	0.00%	100.00%	100.00%
24 h	UVE	LS-SVM	100.00%	93.33%	100.00%	81.81%	100.00%	100.00%
24 h	SPA	PLSR	68.33%	70.00%	5.3%	97.56%	18.18%	100.00%
24 h	SPA	LS-SVM	76.67%	73.33%	47.37%	81.82%	90.24%	68.42%

**Table 5:** Prediction of time after compression damage.

Damage Level	Variable	Calibration	Calibration			Prediction				AB_RMSE
			Rc	Rc^2^	RMSEC	Rp	Rp^2^	RMSEP	RPD	
all	all	PLSR	0.687	0.472	6.504	0.364	0.030	6.834	1.073	0.330
5 mm	all	PLSR	0.491	0.242	7.932	0.401	−0.066	6.628	1.062	1.304
15 mm	all	PLSR	0.731	0.534	6.038	0.868	0.715	3.985	1.959	2.053
all	all	LS-SVM	0.786	0.598	5.671	0.467	0.170	6.532	1.122	0.861
5 mm	all	LS-SVM	0.888	0.755	4.512	0.193	−0.215	7.306	0.964	2.794
15 mm	all	LS-SVM	0.923	0.832	3.622	0.890	0.753	3.693	2.114	0.071
15 mm	UVE	PLSR	0.761	0.579	5.740	0.798	0.580	4.918	1.587	0.822
15 mm	UVE	LS-SVM	0.880	0.745	4.461	0.898	0.770	3.655	2.136	0.806
15 mm	SPA	PLSR	0.621	0.386	6.929	0.860	0.618	4.451	1.754	2.478
15 mm	SPA	LS-SVM	0.681	0.459	6.501	0.867	0.653	4.095	1.907	2.406

## Supplementary Materials

The following are available online at https://www.mdpi.com/1424-8220/20/7/1866/s1, Figure S1: Electronic nose (e-nose) and gas chromatography-mass spectrophotometry (GC–MS) spectral examples of yellow peach from three groups at 4, 8, and 24 h after the fruit was compressed. 0 mm: fruits without compression damage (Group 0), 5 mm: fruits compressed by 5 mm (Group I), 15 mm: fruits compressed by 15 mm (Group II).
