# Supplementary material for: Rapid and Non-Destructive Detection of Compression Damage of Yellow Peach Using an Electronic Nose and Chemometrics
Source: Sensors (Basel). 2020 Mar 27;20(7):1866. doi: 10.3390/s20071866 (PMC7181052; doi:10.3390/s20071866)
Supplement: Supplementary file 1 [file sensors-20-01866-s001.pdf]

sensors

Supplementary Materials

Rapid and Non-Destructive Detection of Compression Damage of Yellow Peach Using Electronic Nose and Chemometrics

Xiangzheng Yang [1], Jiahui Chen [2], Lianwen Jia [1], Wangqing Yu [1], Da Wang [1], Wenwen Wei [1], Shaojia Li [2], Shiyi Tian [3] and Di Wu [2,*]

1 Jinan Fruit Research Institute, All China Federation of Supply and Marketing Cooperatives, Jinan 250014, China; yangxiangzheng318@163.com (X.Y.); lianwejia@163.com (L.J.); jnbxzx@163.com (W.Y.); wangda19910@163.com (D.W.); flying200807@163.com (W.W.)

2 College of Agriculture & Biotechnology, Zhejiang University, Zijingang Campus, Hangzhou 310058, China; 21816131@zju.edu.cn (J.C.); shaojiali@zju.edu.cn (S.L.)

3 School of Food Science and Biotechnology, Zhejiang GongShang University, Hangzhou 310018, Zhejiang, China; tianshiyi@zjgsu.edu.cn

* Corresponding author: di_wu@zju.edu.cn; Tel: +86 571 88982226. Fax: +86 571 88982224

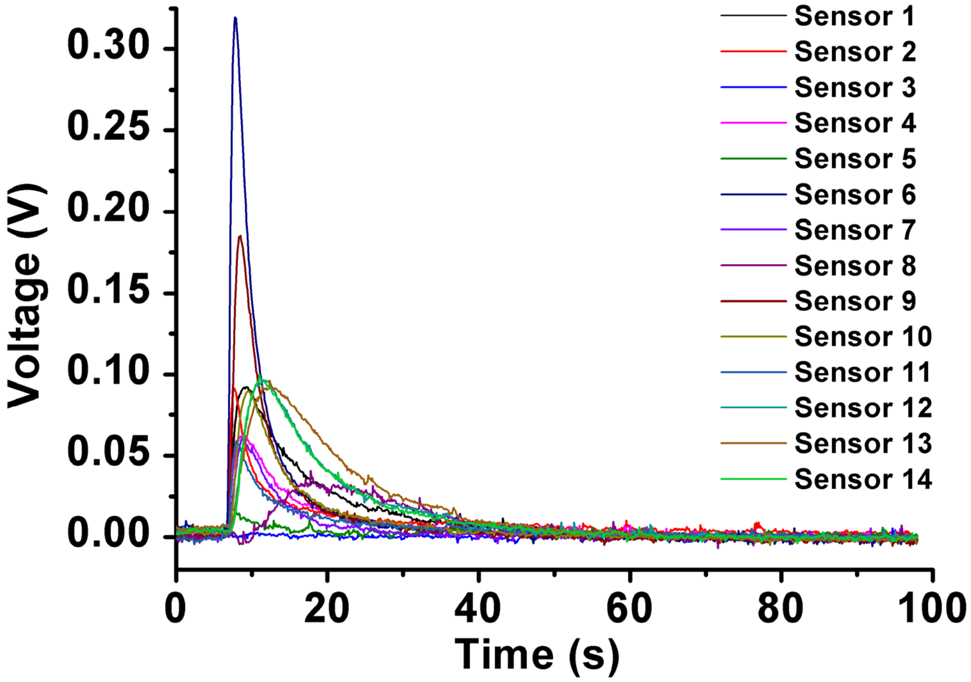

(a) E-nose spectra (4 h-0 mm).

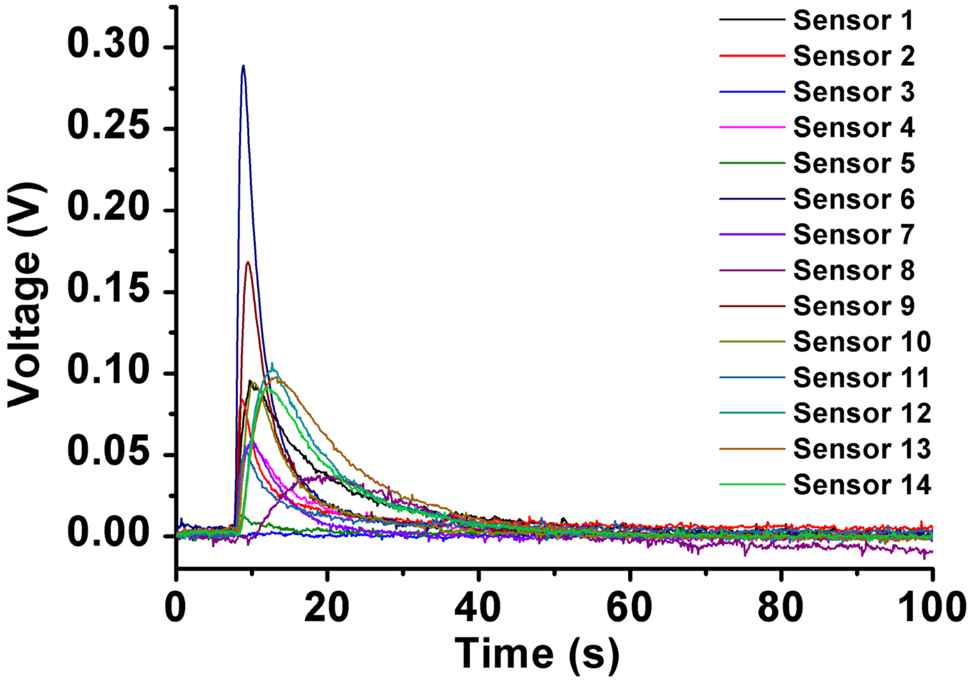

(b) E-nose spectra (8 h-0 mm).

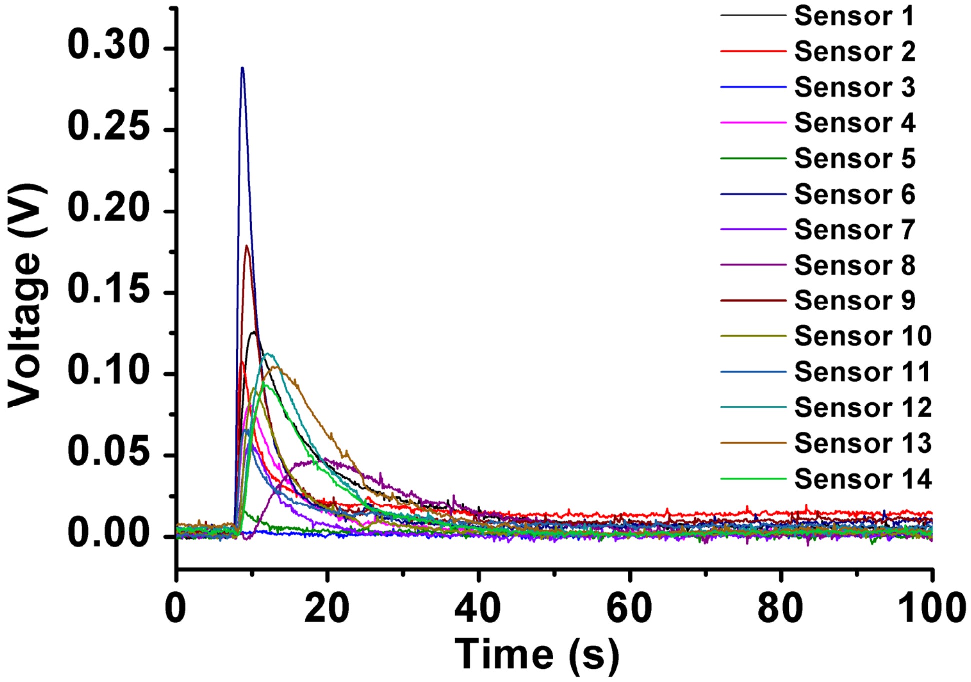

(c) E-nose spectra (24 h-0 mm).

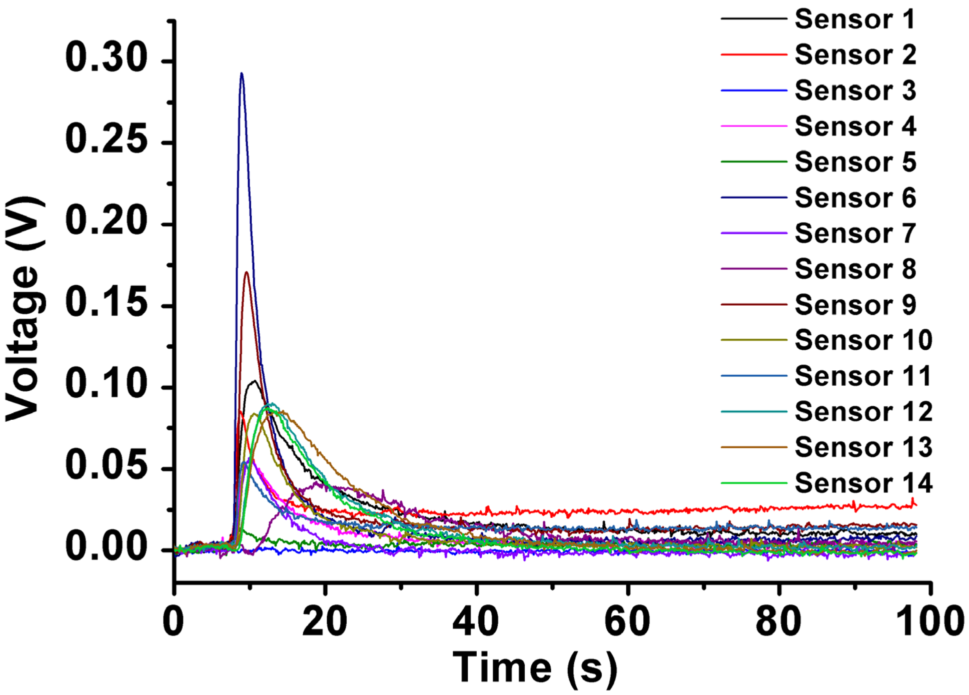

(d) E-nose spectra (4 h-5 mm).

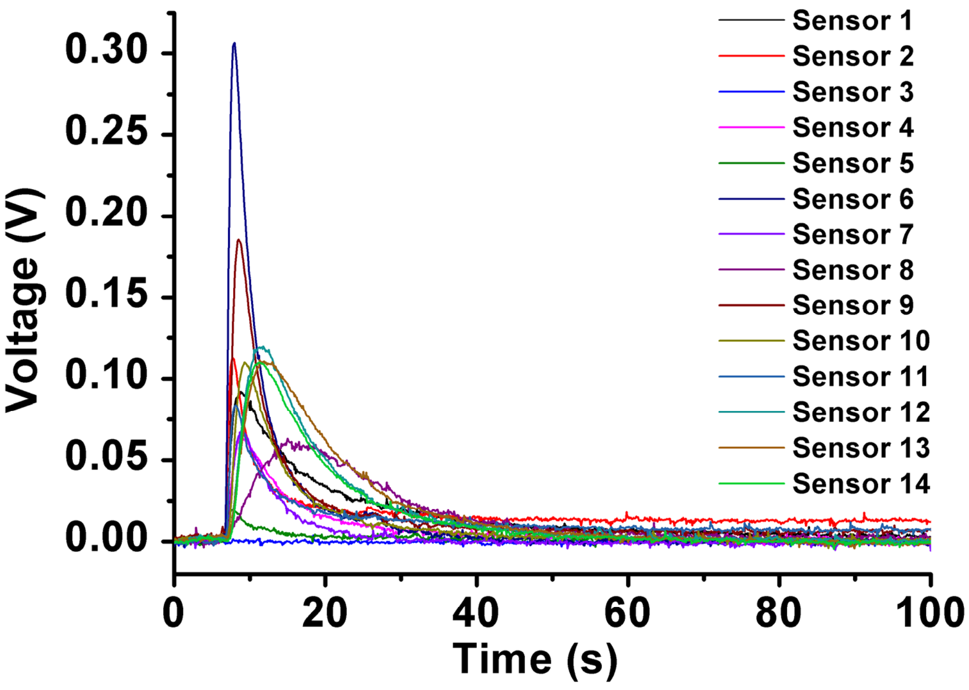

(e) E-nose spectra (8 h-5 mm).

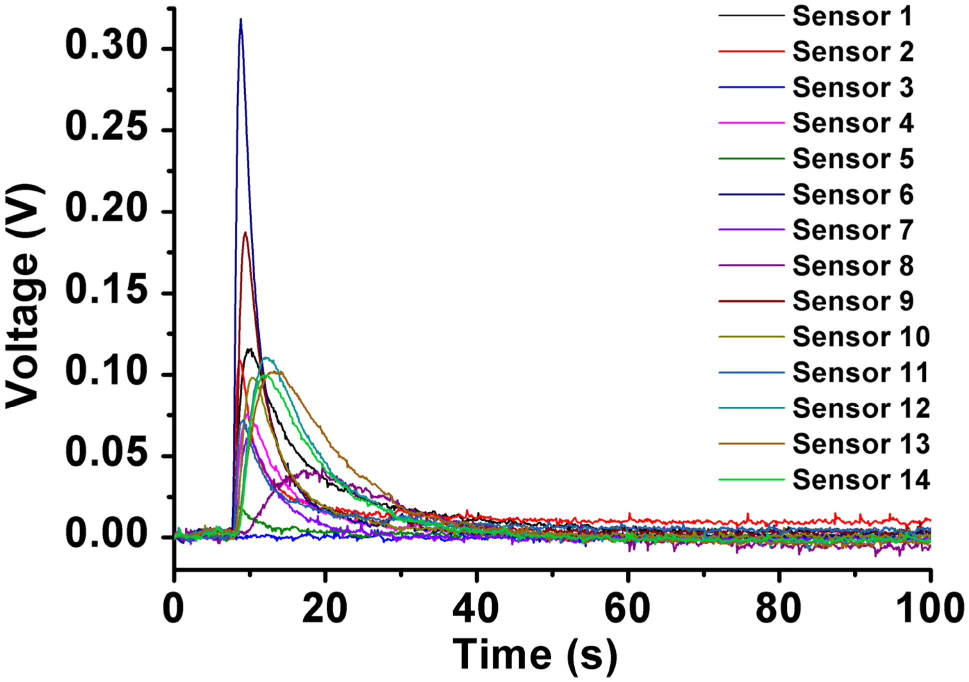

(f) E-nose spectra (24 h-5 mm).

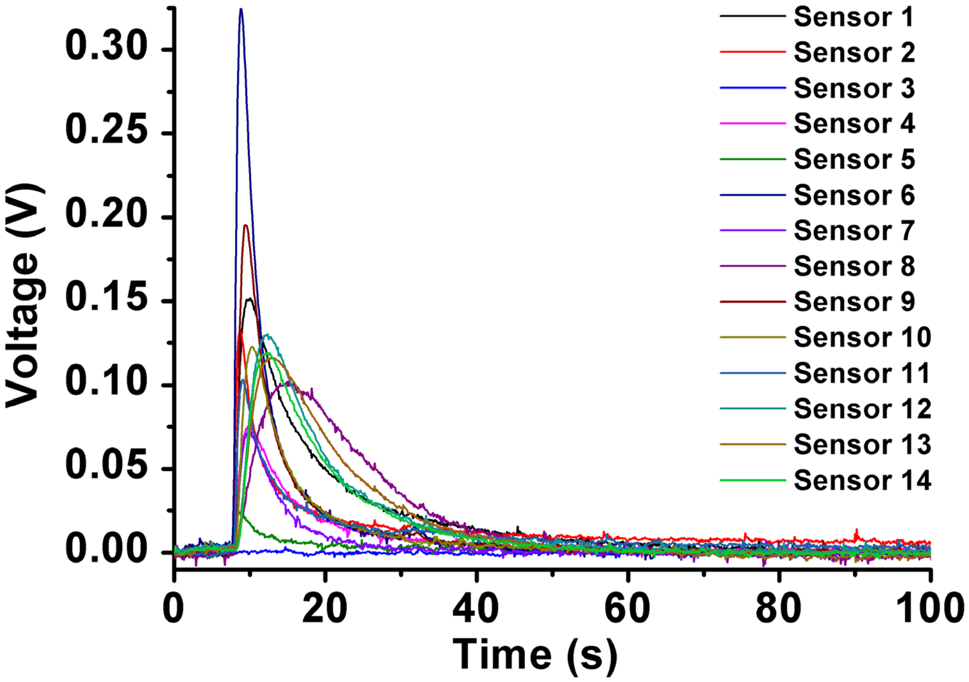

(g) E-nose spectra (4 h-15 mm).

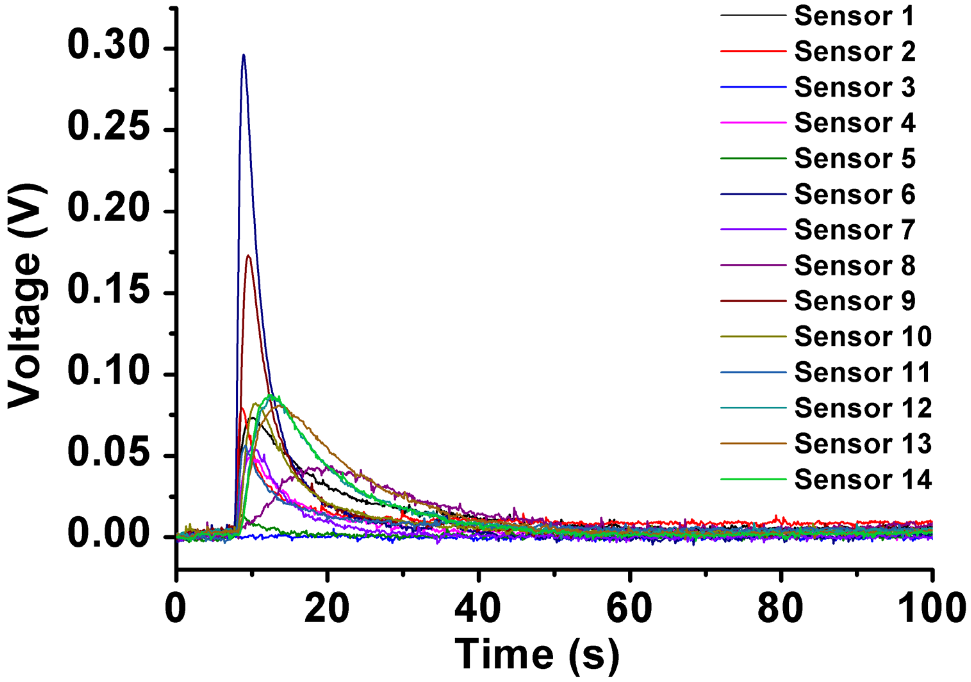

(h) E-nose spectra (8 h-15 mm).

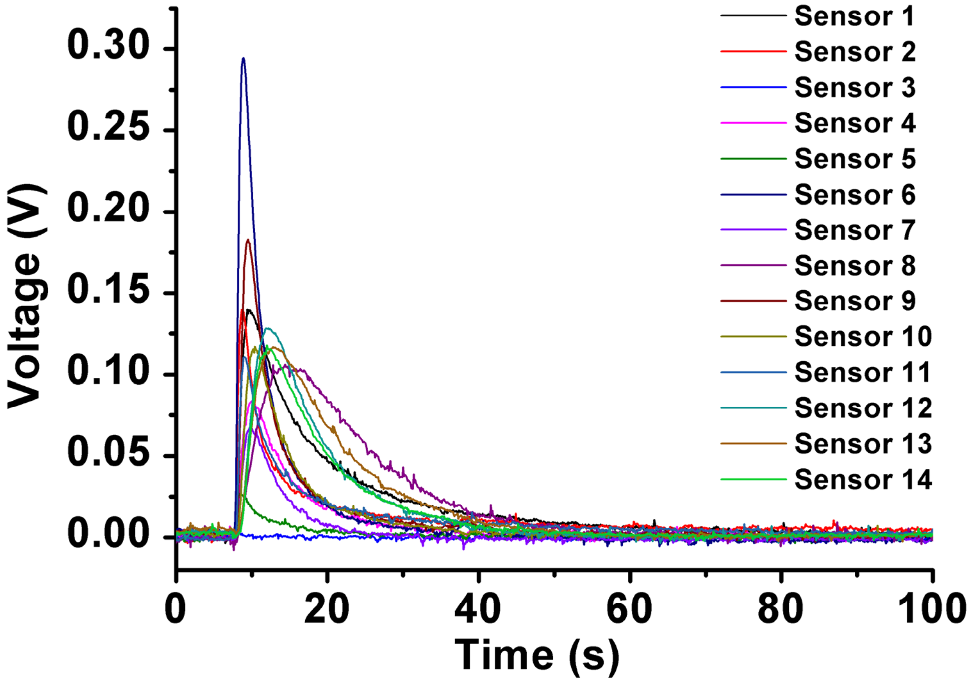

(i) E-nose spectra (24 h-15 mm).

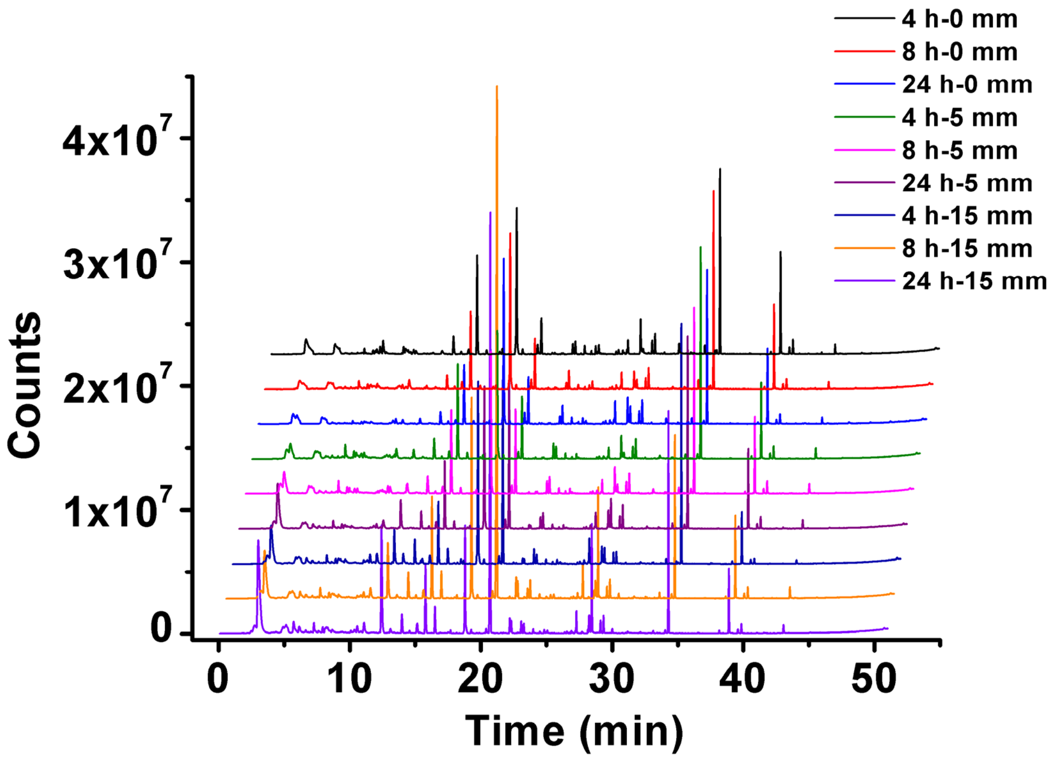

(j) GC-MS spectra.

Figure S1. Electronic nose (e-nose) and gas chromatography-mass spectrophotometry (GC–MS) spectral examples of yellow peach from three groups at 4, 8, and 24 h after the fruit was compressed. 0 mm: fruits without compression damage (Group 0), 5 mm: fruits compressed by 5 mm (Group I), 15 mm: fruits compressed by 15 mm (Group II).
